# Supplementary material for: Recyclable soft photonic crystal film with overall improved circularly polarized luminescence
Source: Nat Commun. 2023 Sep 30;14:6123. doi: 10.1038/s41467-023-41884-5 (PMC10542380; doi:10.1038/s41467-023-41884-5)
Supplement: Supplementary file 1 — Supplementary Information [file 41467_2023_41884_MOESM1_ESM.pdf]

Supplementary Information

Recyclable soft photonic crystal film with overall improved circularly polarized luminescence

Yonghong Shi[1,2], Jianlei Han[1], Chengxi Li[1,2], Tonghan Zhao[1], Xue Jin[1] and Pengfei Duan[1,2]*

[1] CAS Key Laboratory of Nanosystem and Hierarchical Fabrication, National Center for Nanoscience and Technology (NCNST), No. 11 ZhongGuanCun BeiYiTiao, 100190 Beijing, P. R. China.

[2] University of Chinese Academy of Sciences, Beijing 100049, P. R. China.

Correspondence and requests for materials should be addressed to P.D. (duanpf@nanoctr.cn)

Supplementary Table 1 Chemical structures of host LCs, reactive monomers, dye fluorescence, and photoinitiator.

Name	Chemical Structure	Synonyms
**HTG135200**	Commercial Nematic Liquid Crystal hybrid	
**C6M**	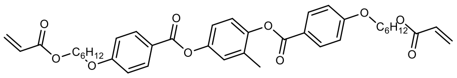	2-methyyl-1,4-phenylene bis(4-((6-(acryloyloxy)hexyl)oxy)benzoate))
**R5011/S5011**	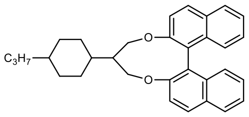	(13bR)-5,6-Dihydro-5-(trans-4-propylcyclohexyl)-4H-dinaphtho[2,1-:1’,2’-h][1,5]dioxonin
**TMPTA**	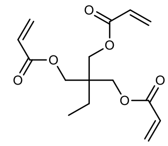	1,1,1-Trimethylolpropane Triacrylate
**I-651**	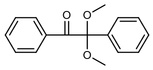	2,2-Dimethoxy-2-phenylacetonphenone
**DPA**	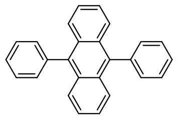	9,10-Diphenylanthracene
**C6**	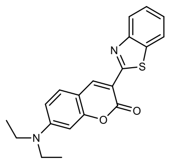	3-(2-Benzothiazolyl)-N,N-diethylumbelliferylamine
**SP**	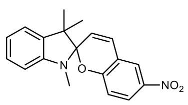	1',3',3'-Trimethyl-6-nitrospiro[chromene-2,2'-indoline]
**TPE**	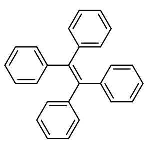	Tetraphenylethylene
**Perylene**	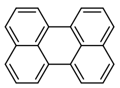	Perylene

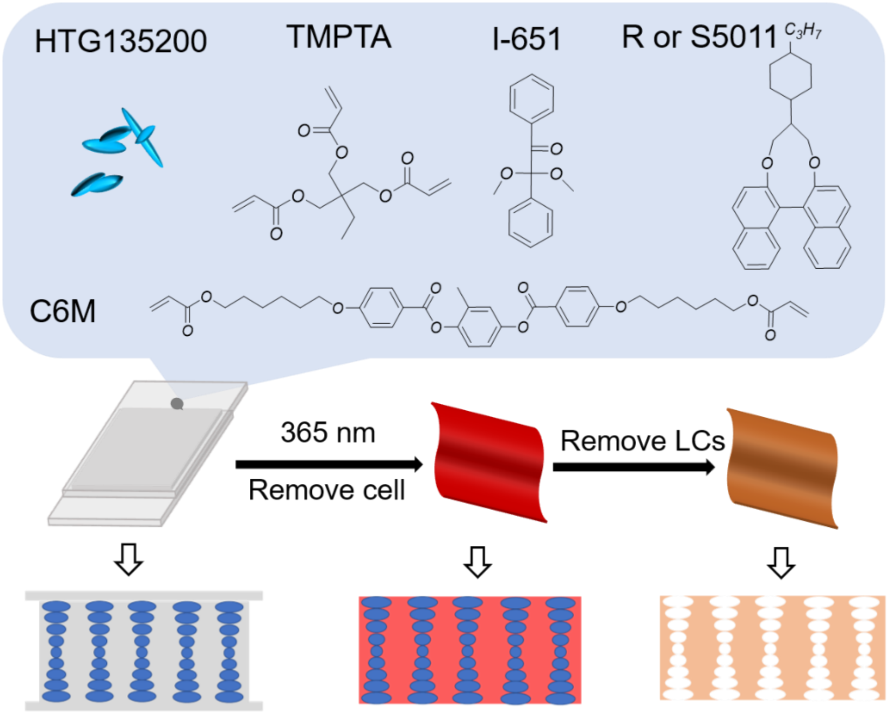

Supplementary Fig. 1 Making process (bottom) and chemical composition (up).

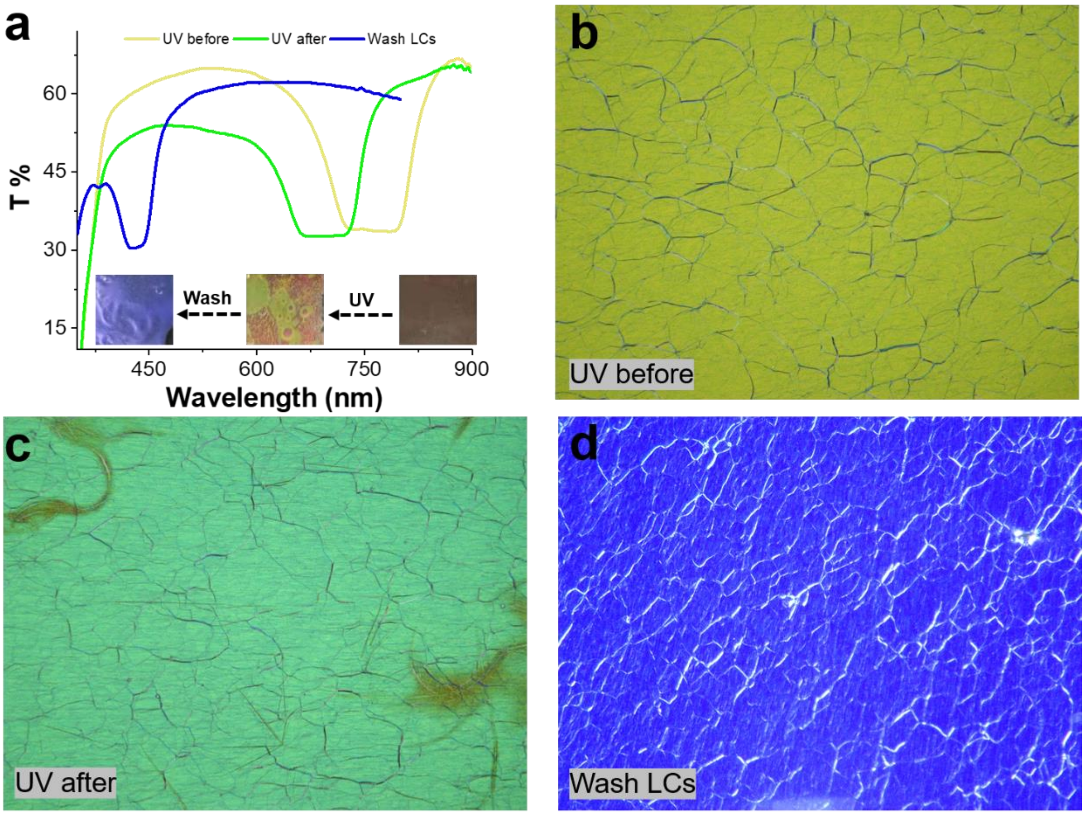

Supplementary Fig. 2 a Transmission spectra of the SPC^S film production process include before

polymerization, after polymerization (UV-365 nm) and remove liquid crystal (the picture in inset). **b-d** Corresponding the planar texture was observed by POM image.

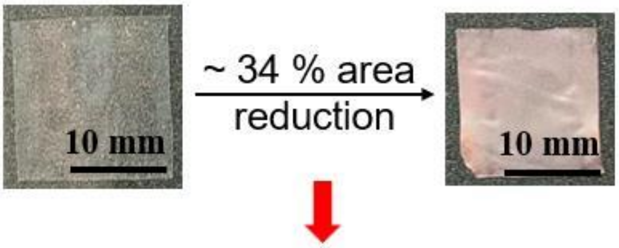

Weight loss percentage	
Theoretical value	Actual average value
63.4 %	64.4 %

Supplementary Fig. 3 Removal of the non-reactive LC led to shrinkage of the polymer network, shortening area to the substrate surface by 34%; The SPC^S lost 64.4% of its weight after washing by analytical balance in laboratory.

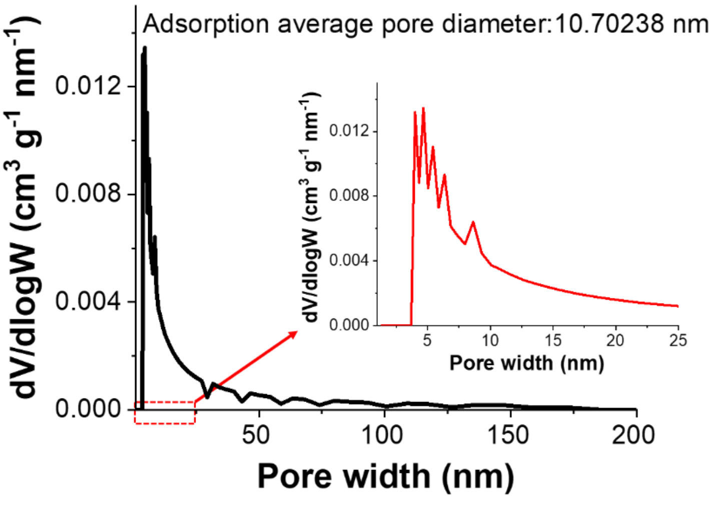

Supplementary Fig. 4 Pore size distributions of chiral SPC film, S5011/HTG135200 = 1.35 wt%.

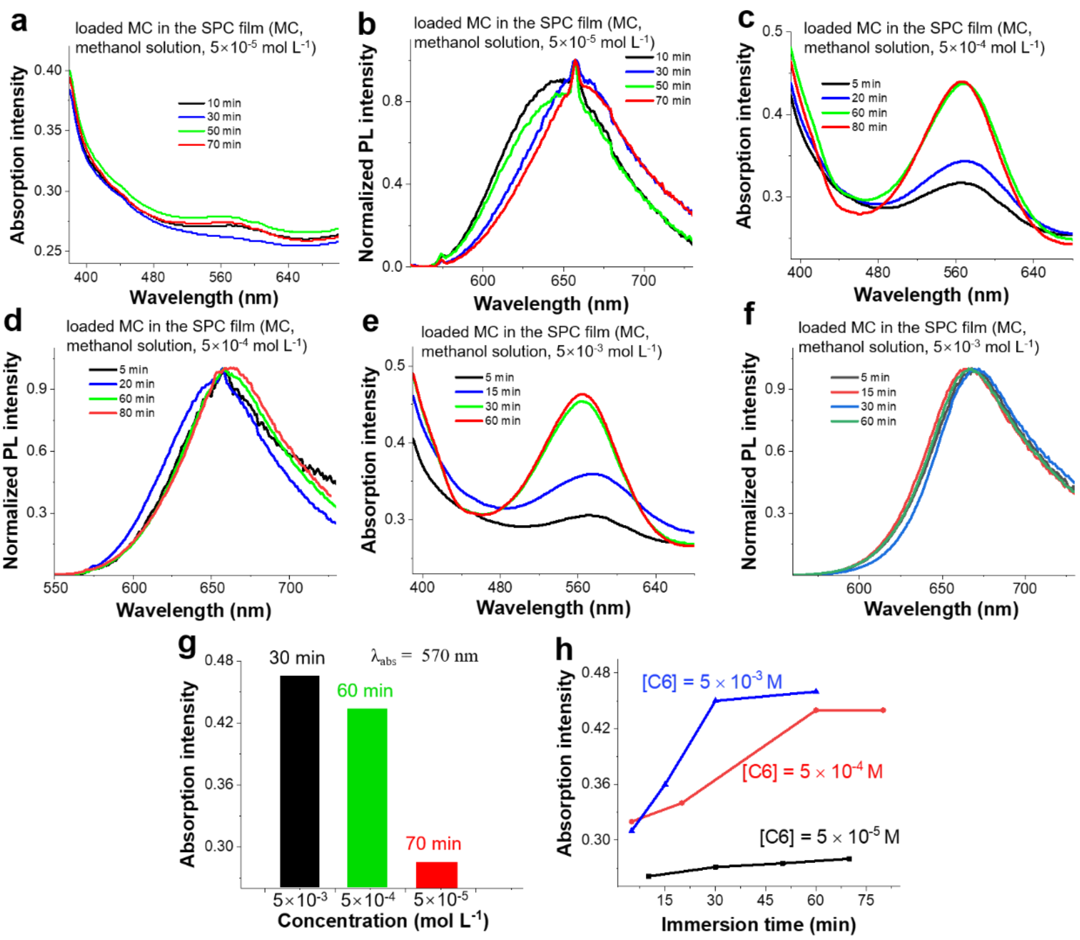

Supplementary Fig. 5 Relationship between soak time and absorption and emission spectra of the chiral SPC films in different concentrations of MC solutions (methanol). **a, b**: [SP] = 5 × 10^{-5} mol L^{-1}, **c, d**: [SP] = 5 × 10^{-4} mol L^{-1}, **e, f**: [SP] = 5 × 10^{-3} mol L^{-1}. **g** The time required for the absorption spectrum to reach saturation at different concentrations. **h** The relationship between soak time and fluorescence emission peak at different concentrations.

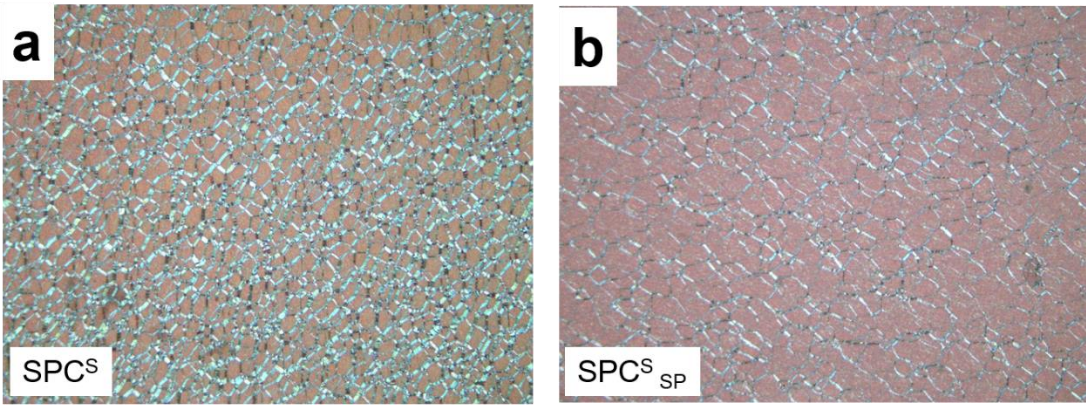

Supplementary Fig. 6 Polarized optical microscopy (POM) images of (**a**) SPC^S and (**b**) $SPC^S{}_{SP}$ film, SPC^S film: S5011/HTG135200 = 1.35 wt%.

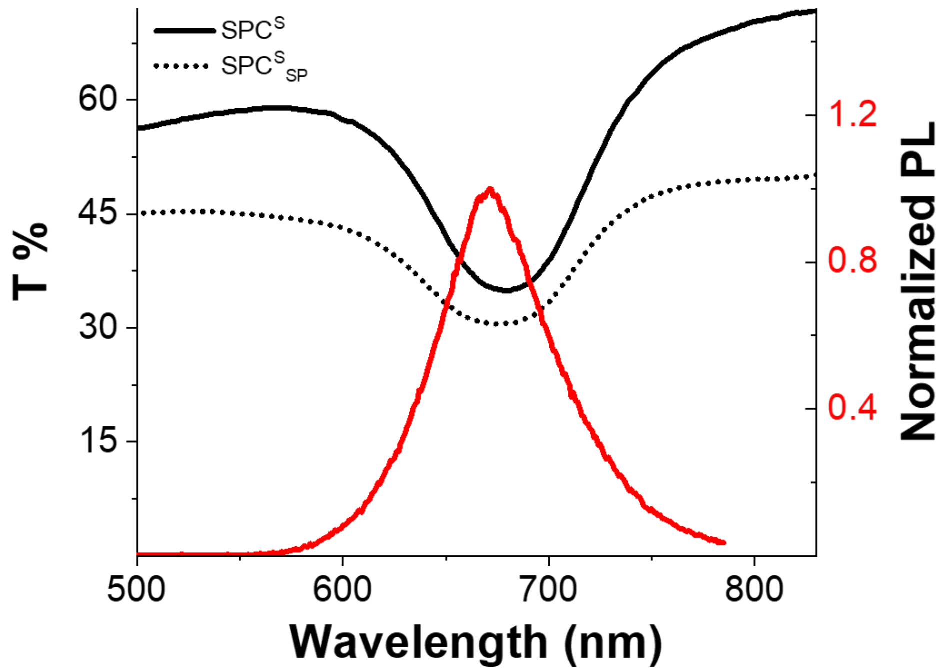

Supplementary Fig. 7 Transmittance spectra of SPC^S and SPC^S $_{SP}$, emission spectra of SPC^S $_{MC}$.

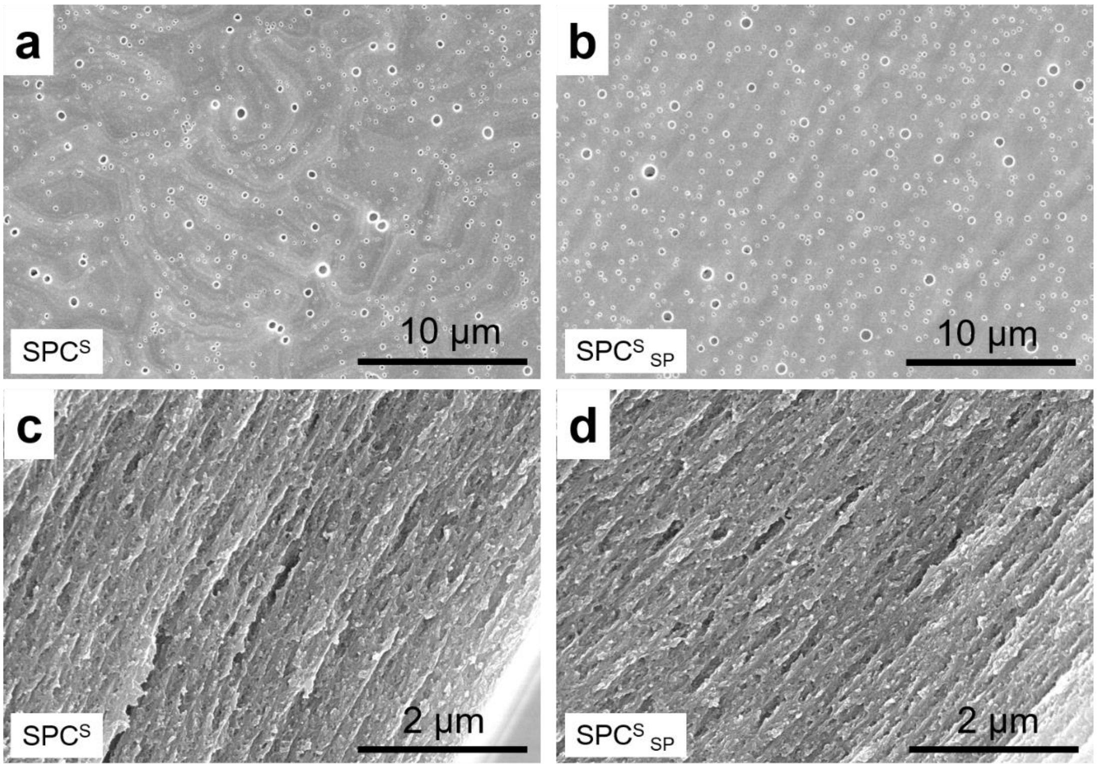

Supplementary Fig. 8 The surface images (**a, b**) and cross section structure (**c, d**) of SPC^S and

$SPC^S{}_{SP}$.

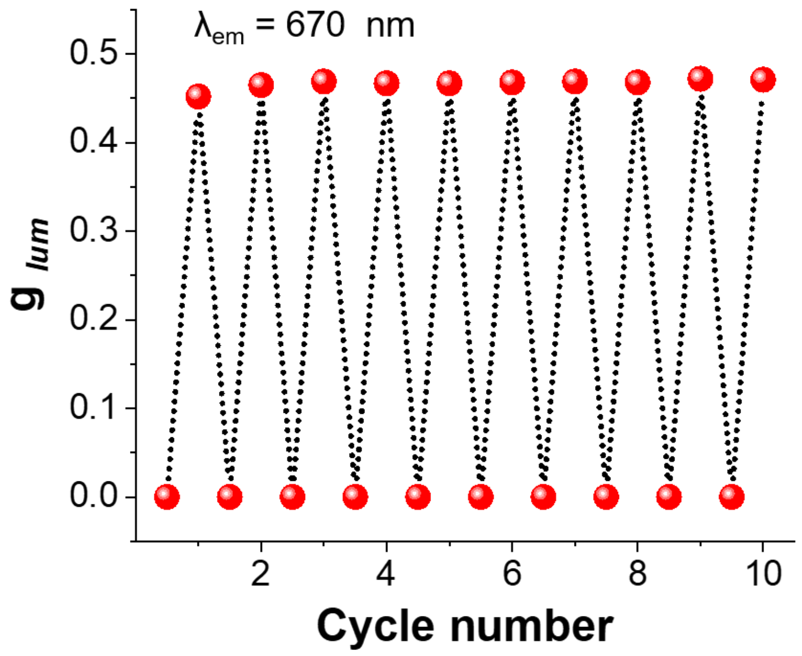

Supplementary Fig. 9 The photoswitching g_{lum} value of the $SPC^S{}_{MC}$ film monitored at 670 nm upon alternating UV light (365 nm) and visible light (465 nm) irradiation.

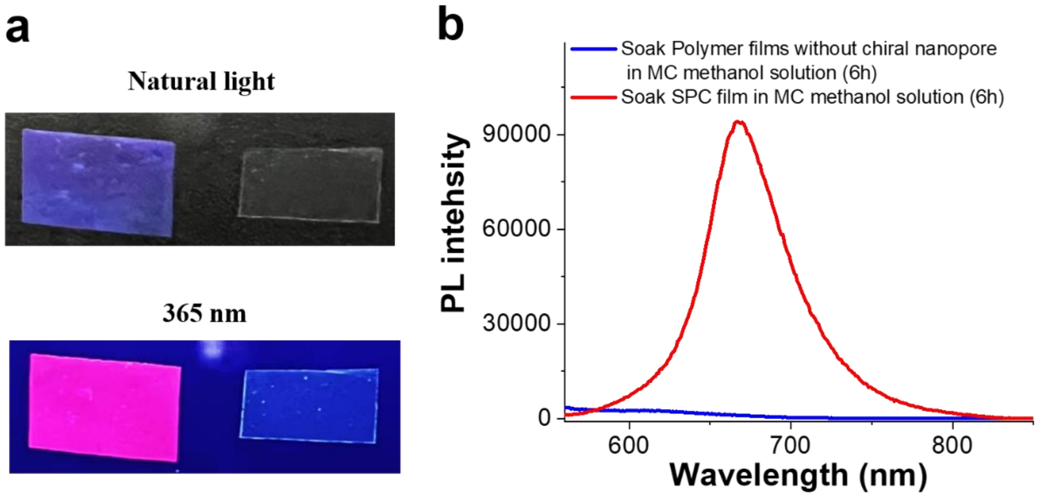

Supplementary Fig. 10 Polymer film without chiral nanopores made from monomers (C6M), initiators (I-651) and crosslinking agents (TMPTA). **a** Photographs of SPC^S film (left) and polymer film without chiral nanopores (right) soaked in spiropyran methanol solution for 6 hours. **b** Corresponding fluorescent spectra.

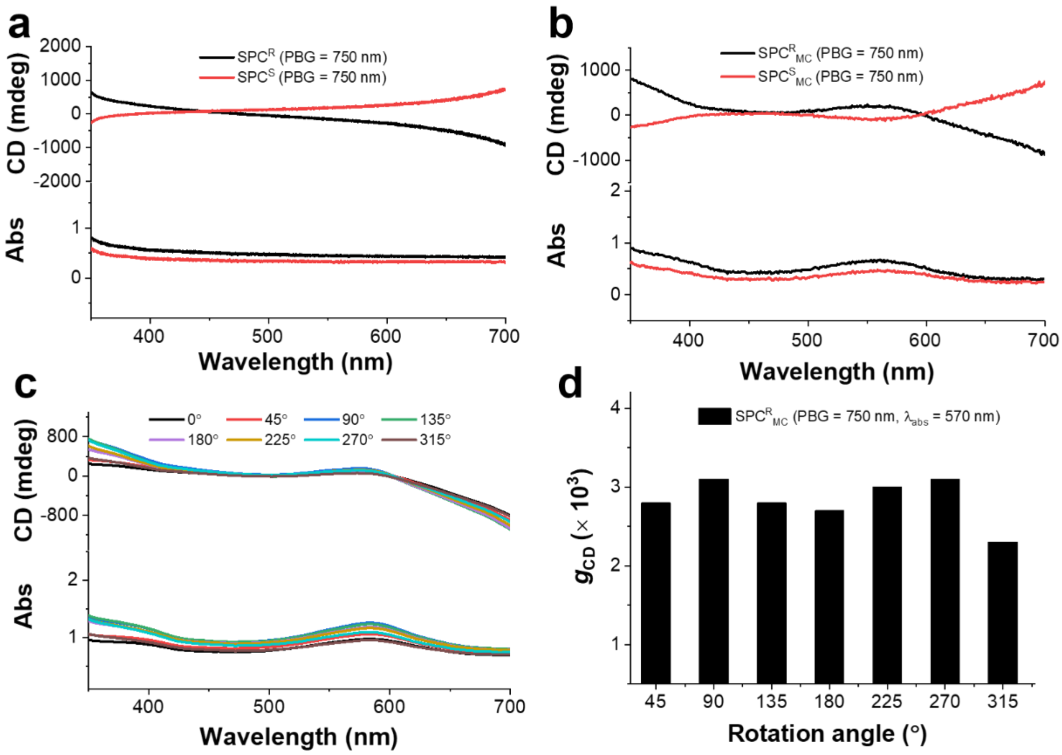

Supplementary Fig. 11 **a** CD spectra of SPC^S and SPC^R films. **b** CD spectra of SPC^S $_{C6}$ and SPC^S $_{C6}$ films. The CD spectra **(c)** and g_{lum} **(d)** of SPC^R $_{C6}$ by changing the angle of the sample along the direction of incident light propagation. SPC^S (or SPC^R) film at weight ratios of S5011 (or R5011)/HTG135200 = 1.1 wt%.

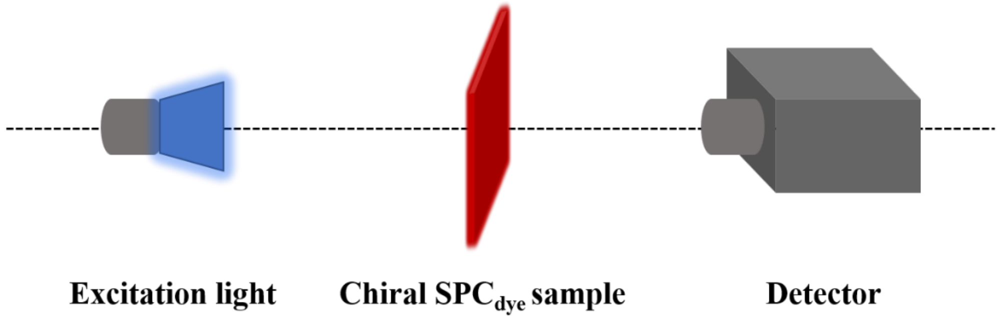

Supplementary Fig. 12 Schematic representation of experimental setup for PL and CPL spectra.

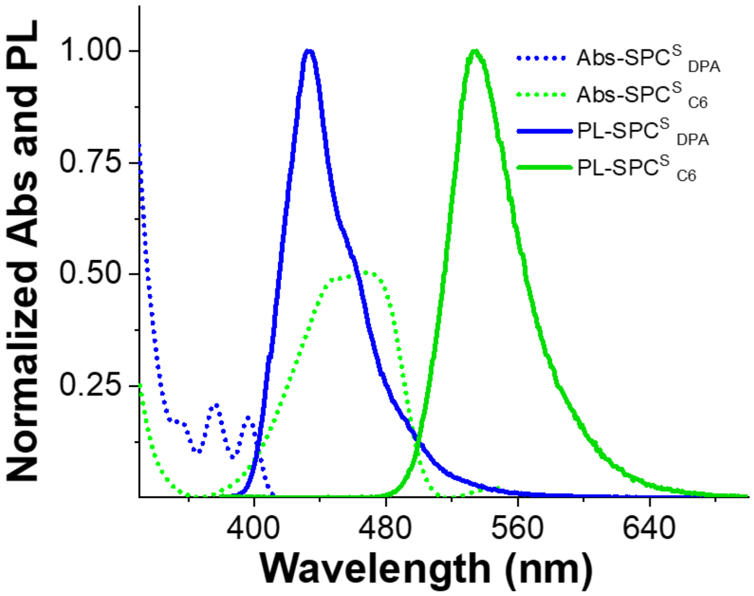

Supplementary Fig. 13 Normalized absorption and fluorescent spectra of SPC^{S} $_{DPA}$ and SPC^{S} $_{C6}$ film (λ_{ex} = 360 nm). SPC^{S} $_{DPA}$ film: S5011/HTG135200 = 2.3 wt%, SPC^{S} $_{C6}$ film: S5011/HTG135200 = 1.9 wt%.

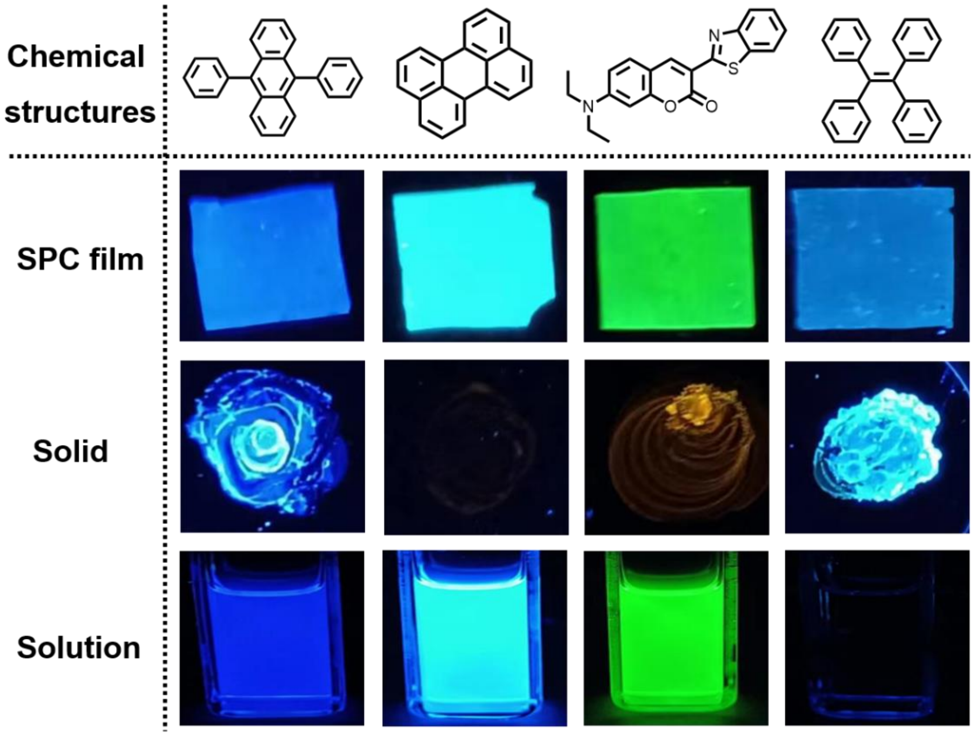

Supplementary Fig. 14 Chemical structures (top) and the fluorescence images of dyes in different states under UV–365 nm light (bottom). The solution concentration of the dye molecules is 10^{-4} mol L^{-1}. SPC^{S} $_{DPA}$ film: S5011/HTG135200 = 2.3 wt%, SPC^{S} $_{C6}$ film: S5011/HTG135200 = 1.9 wt%, SPC^{S} $_{TPE}$ film: S5011/HTG135200 = 2.4 wt%, SPC^{S} $_{perylene}$ film: S5011/HTG135200 = 2.25 wt%.

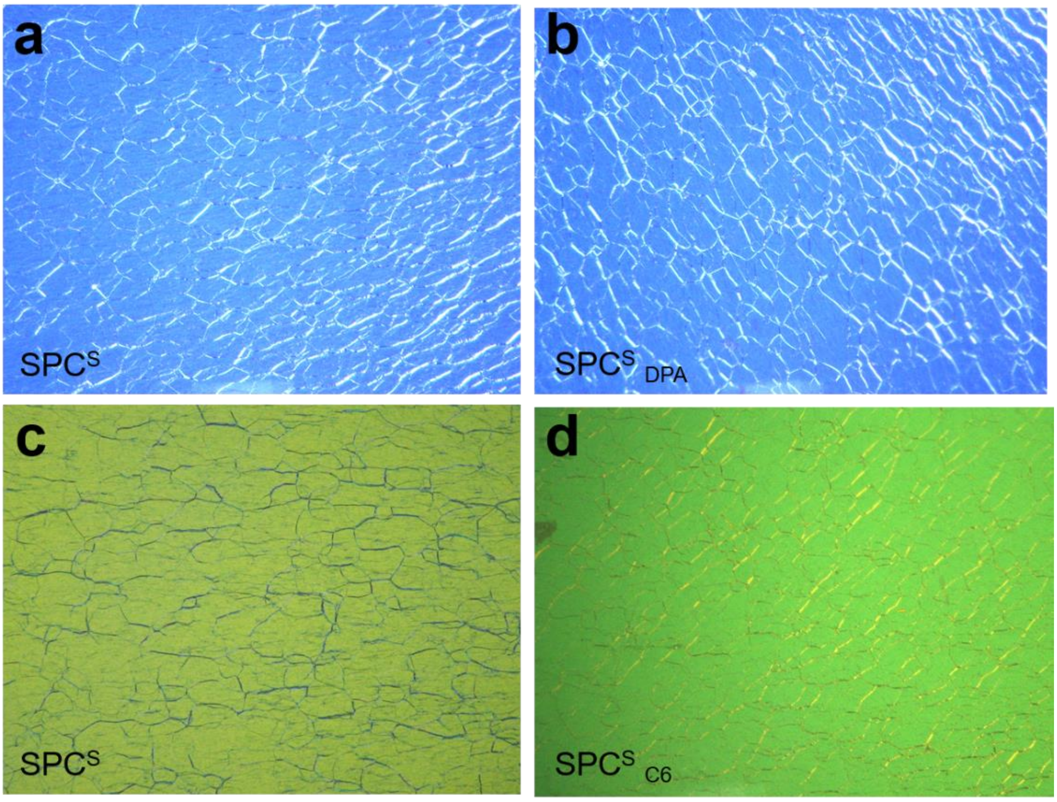

Supplementary Fig. 15 The planar texture of SPC^{S} before (a, c) and after (b, d) the introduction of DPA (SPC^{S} $_{DPA}$, S5011/HTG135200 = 2.3 wt%) and C6 (SPC^{S} $_{C6}$, S5011/HTG135200 = 1.9 wt%).

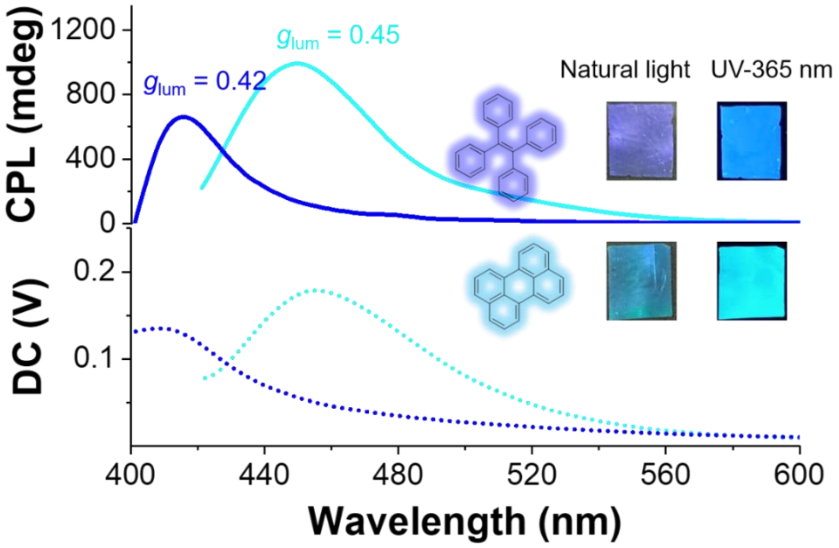

Supplementary Fig. 16 CPL spectral of SPC^{S} $_{TPE}$ (λ_{em} = 415 nm) and SPC^{S} $_{Perylene}$ (λ_{em} = 456 nm) film, λ_{ex} = 360 nm. SPC^{S} $_{TPE}$ film: S5011/HTG135200 = 2.4 wt%, SPC^{S} $_{perylene}$ film:

S5011/HTG135200 = 2.25 wt%.

Supplementary Table 2 Dissymmetry factors (g_{lum}) and absolute fluorescent quantum yield (Φ) of SPC^{S}_{dye} film.

	**SPC^{S}_{MC} (λ_{em} = 670 nm)**	**SPC^{S}_{DPA} (λ_{em} = 430 nm)**	**SPC^{S}_{C6} (λ_{em} = 530 nm)**	**SPC^{S}_{TPE} (λ_{em} = 415 nm)**	**$SPC^{S}_{Perylene}$ (λ_{em} = 456 nm)**
**Φ (%)**	22.32	99.42	75.16	32.9	83.98
**g_{lum}**	0.47	0.43	0.44	0.42	0.45

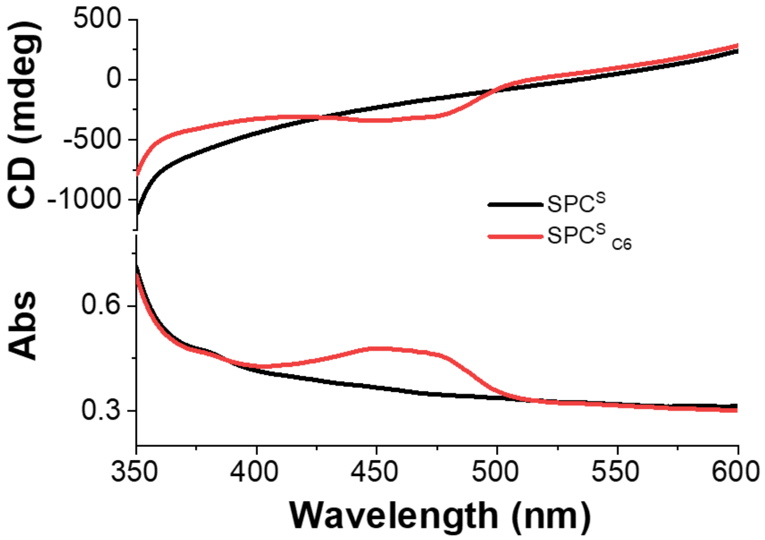

Supplementary Fig. 17 CD spectra of SPC^{S} and SPC^{S}_{C6} films, SPC^{S} (or SPC^{R}) film at weight ratios of S5011 (or R5011)/HTG135200 = 1.1 wt%.

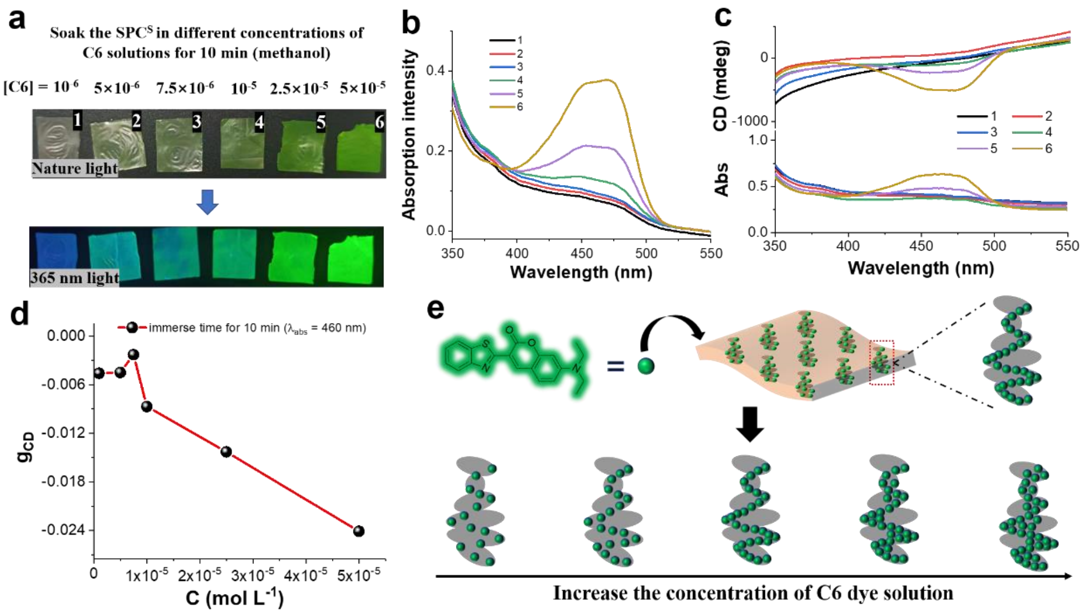

Supplementary Fig. 18 a SPC^S was immersed in solutions of C6 dyes of different concentrations for 10 min and then dried, and image of SPC^S $_{C6}$ were photographed the under the nature light (top) and 365 UV light (bottom). The absorption (**b**) and CD spectra (**c**) of different SPC^S $_{C6}$ film. **d** Concentrations-dependent CD spectra of SPC^S $_{C6}$. **e** Schematic representation of the adsorption process of dye molecules in chiral nanopores imprint of SPC film. As the embedded dye molecules increase, they adsorb orderly into the chiral nanopores imprint. SPC^S (or SPC^R) film at weight ratios of S5011 (or R5011)/HTG135200 = 1.1 wt%.

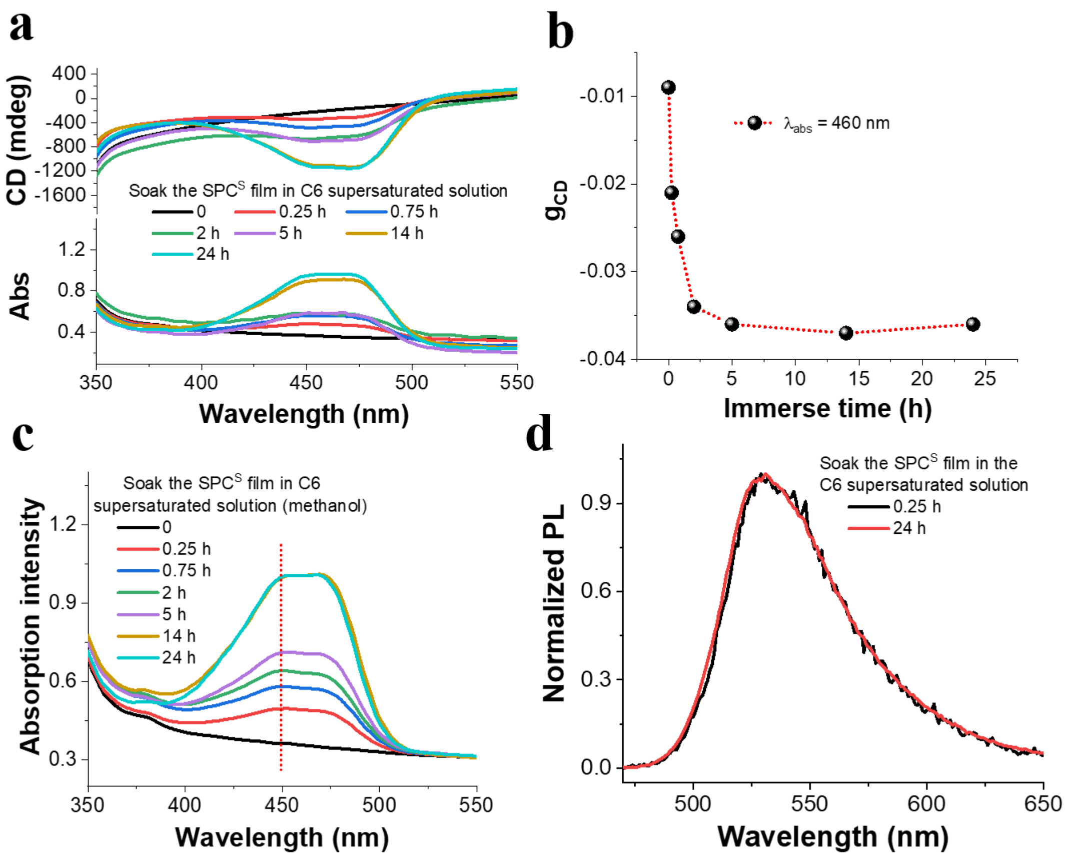

Supplementary Fig. 19 a CD spectra of SPC^S film after immersion in supersaturated solutions of C6 dyes for different times. **b** Immerse time-dependent CD signal intensity of SPC$^S{}_{C6}$ film at 460 nm in supersaturated solutions of C6 dyes. **c** Absorption spectra of SPC^S film after immersion in supersaturated solutions of C6 dyes for different times. **d** Emission spectra of SPC^S film after immersion in supersaturated solutions of C6 dyes for 0.25 h and 24 h. SPC^S (or SPC^R) film at weight ratios of S5011 (or R5011)/HTG135200 = 1.1 wt%.

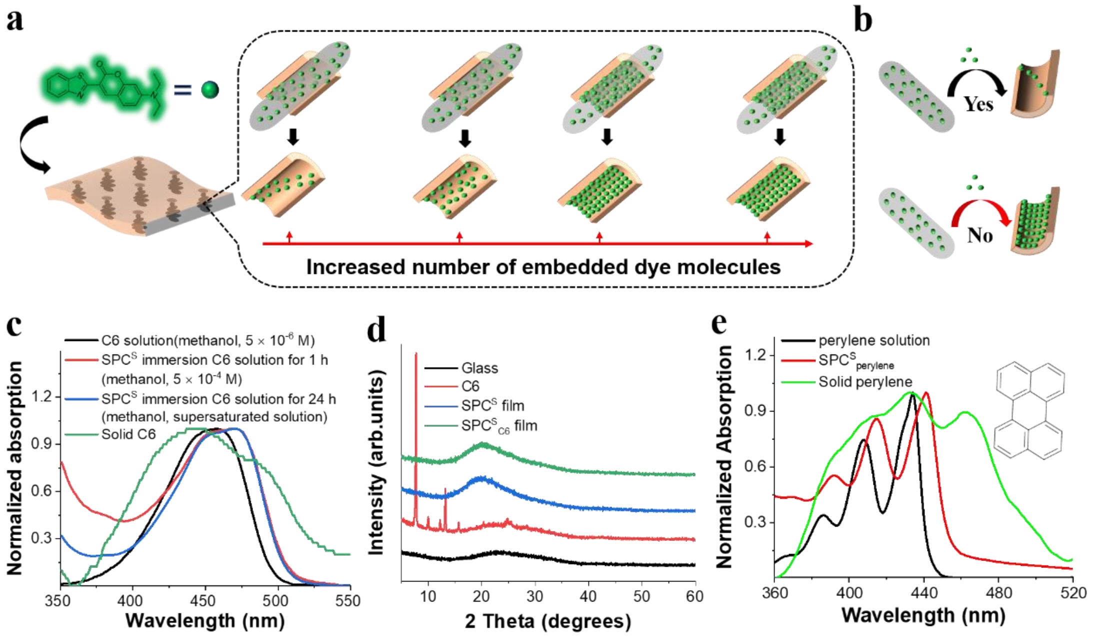

Supplementary Fig. 20 a, b Illustration of the adsorption process of dye molecules in chiral nanopores imprint of the SPC film. **c** Absorption spectra of C6 in solution, SPC^S film and solid state, respectively. **d** XRD spectra of glass, C6, SPC^S, and $SPC^S{}_{C6}$. **e** Absorption spectra of perylene in solution ([Perylene] = 2×10^{-5} mol L^{-1}), SPC^S film and solid state, respectively. The PBG of SPC^S film was 750 nm to eliminate the interference of PBG, SPC^S film: S5011/HTG135200 = 1.1 wt%.

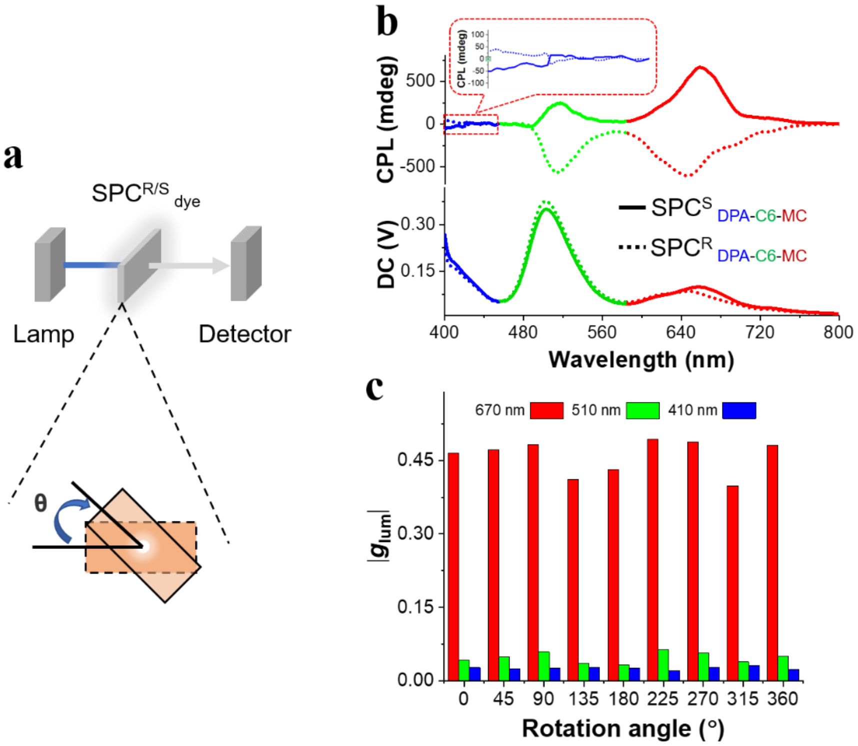

Supplementary Fig. 21 a Schematic representation of experimental setup for CPL spectra. **b** CPL spectra of $SPC^{S}{}_{DPA\text{-}C6\text{-}MC}$ and $SPC^{R}{}_{DPA\text{-}C6\text{-}MC}$ (λ_{ex} = 360 nm). **c** The g_{lum} of $SPC^{S}{}_{DPA\text{-}C6\text{-}MC}$ by changing the angle of the sample along the direction of incident light propagation.
